# Improving neuro-oncological patients care: basic and practical concepts for nurse specialist in neuro-rehabilitation

**DOI:** 10.1186/1756-9966-31-82

**Published:** 2012-10-02

**Authors:** Michelangelo Bartolo, Chiara Zucchella, Andrea Pace, Alessandro Marco De Nunzio, Mariano Serrao, Giorgio Sandrini, Francesco Pierelli

**Affiliations:** 1 NeuroRehabilitation Unit, IRCCS NEUROMED, Mediterranean Neurological Institute, Pozzilli, Isernia, Italy; 2 Palliative Home-Care Unit for Brain Tumors Patients, Regina Elena National Cancer Institute, Rome, Italy; 3 Neurorehabilitation Unit, “Sapienza” University of Rome - Polo Pontino, Latina, Italy; 4 Rehabilitation Centre, Policlinico Italia, Rome, Italy; 5 Neurorehabilitation Unit, IRCCS National Neurological Institute C. Mondino Foundation, Pavia, Italy; 6 Department of Neurological Sciences, University of Pavia, Pavia, Italy; 7 Department of Medico-Surgical Sciences and Biotechnologies, “Sapienza” University of Rome, Rome, Italy

**Keywords:** Neurorehabilitation, Nurses, Education, Certification, Core curriculum, Team, Brain tumors

## Abstract

**Background:**

Neuro-oncological population well expresses the complexity of neurological disability due to the multiple neurological deficits that affect these patients. Moreover, due to the therapeutical opportunities survival times for patients with brain tumor have increased and more of these patients require rehabilitation care. The figure of nurse in the interdisciplinary specialty of neurorehabilitation is not clearly defined, even if their role in this setting is recognized as being critical and is expanding.

The purpose of the study is to identify the standard competencies for neurorehabilitation nurses that could be taught by means of a specialization course.

**Methods:**

A literature review was conducted with preference given to works published between January 2000 and December 2008 in English. The search strategy identified 523 non-duplicated references of which 271 titles were considered relevant. After reviewing the abstracts, 147 papers were selected and made available to a group of healthcare professionals who were requested to classify them in few conceptual main areas defining the relative topics.

**Results:**

The following five main areas were identified: clinical aspects of nursing; nursing techniques; nursing methodology; relational and organisational models; legal aspects of nursing. The relative topics were included within each area. As educational method a structured course based on lectures and practical sessions was designed. Also multi-choices questions were developed in order to evaluate the participants’ level of knowledge, while a semi-structured interview was prepared to investigate students’ satisfaction.

**Conclusions:**

Literature shows that the development of rehabilitation depends on the improvement of scientific and practical knowledge of health care professionals. This structured training course could be incorporated into undergraduate nursing education programmes and also be inserted into continuing education programmes for graduate nurses. Developing expertise in neuro-rehabilitation for nurses, will be critical to improve overall care and care management of patients with highly complex disabilities as patients affected by brain tumors. The next step will be to start discussing, at the level of scientific societies linked to the field of neurorehabilitation and oncology, the development of a specialisation course in neurorehabilitation nursing.

## Background

Neurological damage is the underlying cause of disability in around 40% of the most severely disabled people (who require daily help), and in the majority of people with complex disabilities involving a combination of physical, cognitive and behavioural impairments [1,2].

The complexity of neurological disability is well represented by neuro-oncological population: in the course of the disease, in fact, patients affected by malignant brain tumor (BT) present multiple neurological deficits, due to primary tumor effects and the adverse effects of treatments that pose important limitations to patient’s everyday functioning [3].

Impaired cognition, weakness, visuo-perceptual and motor problems were the most common neurological deficits reported in the population of patients with BTs [4]. Because of the recent advances in surgical techniques, chemotherapy, and radiation therapy, survival times for patients with BTs have increased and more of these patients require rehabilitation support and services [5-8]. In fact, when cancer is viewed as a chronic disease, the concept of cancer rehabilitation become an important aspect of comprehensive care: patients not only expect physical rehabilitation, but also a broad range of services offered to develop skills which can enable them to cope with the long term consequences of cancer diseases [9,10]. For this reason provision of individual- and group-oriented rehabilitation programs satisfies the patients’ demands for continuity in care and for encouragement to develop self-management skills as described in the Chronic Care Model of the World Health Organization (WHO) [11].

Rehabilitation intervention in cancer patients is recommended both in early stage of disease, for restoring function after surgery and cancer therapy, and in advanced stage of disease as important part of palliative care with the aim to prevent complication, control the symptoms and maintain patients’ independence and quality of life [12-16].

In the context of rehabilitation care to disabled neurological patients, nurses play a key role as patients are highly dependent both on them and on healthcare assistants [17].

Rehabilitation nursing practice is a specialty area in which the aim is to help individuals with disabilities and chronic illnesses regain and maintain optimal health, but also to prevent the occurrence of common complications [18].

In the past, the lead for rehabilitation programmes often came from physiotherapists and occupational therapists. The contribution of the nurse to the rehabilitation process has not always been valued or regarded as an equal member of the rehabilitation team [19].

Nurses were expected to assume little more than an understudy’s role, providing the necessary care required by the patient who was preparing for “rehabilitation”. However, much of this care remained invisible and almost absent from the literature [20], despite Henderson [21] proclaiming that nurses were “rehabilitators par excellence”. She recognized that many of the components of nursing care were not so much basic but essential rehabilitation nursing skills such as relieving pain; helping with hygiene and mobilization; giving pressure area care; ensuring adequate nutrition; promoting and managing continence; giving emotional support; providing patients and caregivers education; and providing opportunities for adequate sleep, rest and stimulation. Unless such needs are fully met and built into an educational rehabilitation programme, all other activities are ineffective.

In addition to their clinical role, rehabilitation nurses also have an important administrative function, effectively acting as case managers, especially in acute care and acute rehabilitation settings. In this role, nurses must advocate for patients and families, representing their concerns regarding care both within and outside the clinical setting [22-24]. The case manager must review each patient individually to establish what treatments and services are appropriate. This role is bound to become increasingly important in the context of the ever-increasing need to achieve better management of resources and shorter hospitalizations.

Nurses who are interested in neuro-oncological rehabilitation are concerned with changes and functional abilities, rather than the disease process, and with how to improve the remaining time, rather than with how many months an individual has left to live. As Dietz states, in fact, the goal of rehabilitation for people with cancer is to improve the quality of life for maximum productivity with minimum dependence, regardless of life expectancy [25].

The complexity of knowledge and skills required to provide such comprehensive care to neuro-oncological patients illustrates the need for increasing specialisation within the health professions [26,27].

Although nursing is purportedly about meeting the needs of all, the development of an understanding of patients with disabilities is one area that is generally not given specific attention in undergraduate nursing curricula [28]. Only a third of nurses felt, with hindsight, that their pre-registration education had provided them with adequate skills and knowledge for their role in rehabilitation; furthermore, nurses have expressed the need to have access to more education and training focused on rehabilitation per se and associated clinical skills, in order to strengthen and raise the profile of their professional role [29-31].

In this regard, *The Specialty Practice ofRehabilitation Nursing: A CoreCurriculum*, published by the Association of Rehabilitation Nurses (ARN) is a key text. Designed both for professionals entering rehabilitation nursing and for those already in the field, it is an important resource for those preparing for the Certified Rehabilitation Registered Nurse (CRRN) examination. In short, in the US, it is a fundamental reference guide to rehabilitation nursing [32].

Currently in Europe there is a discrepancy in training courses for nurses, Table 1. Training achieve different titles, as well as different is the duration in years of training. The only unifying element, which dates back to 1977, is represented by the European directives (77/452/EEC and 77/453/EEC, 27 June 1977) that governed the harmonization of programs and the number of hours needed to become nurse: 2300 of theory and 2300 of clinical practice (180 credits - CFU).

**Table 1:** Nursing education in Europe

**Traditional schools**	**Higher Professional Schools**	**Traditional Schools and University**	**University**
France	Holland	United Kingdom	Spain
Germany	Denmark	Ireland	Italy
		Norway	Northern Ireland
			Scotland
			Wales

In Italy, the role of the nursing profession in the interdisciplinary specialty of neurorehabilitation remains poorly defined. There is currently no structured system allowing nurses to undertake further training to become nurse specialists (NSps) or nurse practitioners (NPs) in neurorehabilitation, and there is no system for the validation and accreditation of nursing skills. There therefore exists a need to promote excellence in rehabilitation nursing care by validating specialist knowledge and introducing qualifications in this area.

These needs prompted us to propose a structured pathway that could be followed by staff nurses wishing to become NSps in neurorehabilitation. Specifically, the purposes of this paper are to identify areas of need within nurses’ clinical education and to propose an education course, defining the main topics to be included in a neurorehabilitation nursing core curriculum.

## Methods

A literature review was conducted by means of PubMed, Cochrane database, and web searches for potentially relevant titles combining the search terms “nurses” and “nursing” with “education”, “rehabilitation”, “neurology”, “neuro-oncology”, “brain tumors”, “learning”, “core curriculum”. The main limits applied for the PubMed search were: clinical trial; meta-analysis; practice guideline; review; classical article; consensus development conference, NIH; guideline; journal article; newspaper article; MEDLINE; nursing journals; systematic reviews. Preference was given to works published between January 2000 and December 2008 in English. The search strategy identified 523 non-duplicated references of which 271 titles were considered relevant. After reviewing the abstracts, 147 papers were selected and made available to a group of healthcare professionals (nurses, physicians, physiotherapists, psychologists) with specific experience in neurorehabilitation, to perform a final revision.

Each professional reviewed the articles and identified a limited number of areas and related topics deemed, by them, fundamental for anyone seeking to acquire the knowledge and skills needed to practice rehabilitation nursing.

The results were compared and discussed among the professionals in order to include the identified areas and topics in the course; a consensus level ≥ 60% was requested otherwise the area or the topic were erased.

### Course description

The discussion among the professionals led to the identification of the following five main areas: a) clinical aspects of nursing; b) nursing techniques; c) nursing methodology; d) relational and organisational models; e) legal aspects of nursing. The topics included in each area are listed in Table 2.

**Table 2:** Course areas and topics

		**Hours**
**Area**	**Clinical aspects of nursing**	**16**
**Topics**	The International Classification of Functioning: basic concepts	1
	Functional anatomy of central and peripheral nervous system	1
	Cerebrovascular diseases	2
	Movement disorders	2
	Dementia	2
	Spinal cord injuries. Multiple sclerosis.	2
	Traumatic brain injuries, coma and vegetative state	2
	Functional disorders (neurological bladder, dysphagia). Sleep. Behavioural disorders. Pain.	2
	Neurooncology	2
**Area**	**Nursing techniques**	**16**
**Topics**	Emergency management and Basic Life Support	2
	Nursing of patients in neurorehabilitation	2
	Posture and mobilisation of neurological patients	2
	Prevention and treatment of pressure sores	2
	Management and complications of nasogastric tube and artificial nutritional systems	2
	Management and complications of the central venous catheter	2
	Management and complications of the orotracheal cannula	2
	Nursing management of bladder functions	2
**Area**	**Nursing methodology**	**10**
**Topics**	Identification of the professional profile of nurses	2
	Operational and information tools of nursing (guidelines, protocols, procedures, protocol preparation methods, clinical and functional assessment scales, nursing folder)	2
	Individual rehabilitation project and programme	2
	Establishment of levels of care and necessary aids/assistance. Regulatory framework	2
	Clinical monitoring equipment and rehabilitation technologies relevant to nursing	2
**Area**	**Relational and organisational models**	**8**
**Topics**	Rehabilitation team: roles and professionals involved	2
	Rehabilitation team: mode of activation, development and management	2
	Organisational models of the nursing team and working methods	2
	Models and methods of communication	2
**Area**	**Legal aspects of nursing**	**8**
**Topics**	Health and safety at work (Law 81/08)	4
	Rights and duties of workers	4

These issues have become the contents of a structured course, amounting to a total of 160 hours that includes three modules: theory (58 hours), practice (22 hours) and observation of experienced nurses (80 hours).

The first module, delivered in the form of lectures, focused on theoretical aspects related to the five main areas. In the second and third modules, the participants received supervised practical training and were able to familiarise themselves with the logistics and use of various equipment, with patient management and with intervention protocols. Basic techniques were demonstrated and then applied by all the participants in turn.

The course should last four weeks (6 days/week, 7 hours/day). The mornings will be were devoted to supervised practical activities and observations on the ward, and the afternoons to theoretical lessons. The setting for all these activities should be a highly specialised neurorehabilitation unit.

The course teachers should be physicians (neurologists, an anaesthetist, a physiatrist), nurses, bioengineers, psychologists, and physiotherapists, all with specific experience in field of neurorehabilitation.

The course will end with the presentation of a thesis. Self-administered questionnaires with multiple choice answers and regarding all the topics should be compiled by the participants to assess their basic level of knowledge, learning and satisfaction.

## Discussion

This paper identifies the standard competencies of the neurorehabilitation nurses and describes a proposed structured education course to train specialist nurses in neurorehabilitation care.

To this end, drawing on the expertise of different clinicians and professionals a consensus was reached on a minimum core set of topics which covered five aspects of rehabilitation nursing: clinical, technical, methodological, organisational and legal.

Consistent with previous literature, this review seems to support the need (perceived by nurses themselves) for specific education and training in order to work with people with complex neurological disabilities [33].

Indeed, a wider investigation of the role of nurses within the multiprofessional rehabilitation team revealed gaps in the skills and knowledge of graduate nurses working in rehabilitation settings: while the role of nurses has evolved considerably, there are still obvious gaps in current rehabilitation nursing training [34].

Moreover, the precise role of nurses in rehabilitation is not clearly defined: the literature shows that rehabilitation nursing has developed to various degrees worldwide. Furthermore, no comprehensive framework for the specialty practice of rehabilitation nursing can be found in the English language literature through Medline and Google searches [35].

The proposed course aims to fill these gaps, providing the necessary theoretical and practical bases, to train a professional NSp in neurorehabilitation. Specifically, its main objectives are: (a) to train nurses, providing them with the expertise to manage the care of neurological patients with disabilities, in both the acute and the chronic phase; (b) to provide them with the skills needed to lead and coordinate multidisciplinary teams so as to ensure the comprehensive care of patients; (c) to transfer, to them, knowledge about the clinical tools and technologies adopted within the field of neurorehabilitation; (d) to impart to them a working method that will enable them to go on expanding their knowledge base as well as to pass it on to other care providers, implementing this knowledge throughout the healthcare system, thereby increasing levels of both safety and quality.

The Association of Rehabilitation Nurses in the US has published a series of documents to guide the development of rehabilitation nursing practice – in particular three editions of a core curriculum [36-38]. Outside the US, however, these publications seem to have had limited impact. Elsewhere, there seems to be agreement that the potential role of nurses in rehabilitation is yet to be fully realized [39-49]. To achieve this goal, the development and implementation of formal education courses could be a key strategy, making it possible to train advanced practice nurses, particularly neurorehabilitation specialists, who could fill the growing need for expert clinicians able to assume major leadership roles in clinical, management and research areas. The course proposed in this paper is based on a minimum set of topics, grouped into five main areas, and could serve as a basis for a core curriculum. This model includes extensive clinical practice and focuses strongly on evidence-based practice; moreover, it highlights the importance of cross-disciplinary teaching, which aims to bring together and harmonise different professional skills in an interprofessional education framework [50,51].

Interdisciplinary healthcare teams with members from many professions have to work closely with each other in order to optimise patient care [52]. In this context, non-technical skills such as communication, collaboration, cooperation and reflection are crucial for effective practice. As interprofessional collaboration is an important element in total quality management, education on how to function within a team is essential [53]: healthcare workers with different knowledge and backgrounds have to harmonise their intervention plans according to the competencies and goals of the other team members [54].

This need for integration is even greater for neuro-oncological patients in which the clinical complexity that derives from the coexistence of disability at different levels, requires a coordinated and synergistic intervention. Based on the bio-psycho-social model of the WHO and a holistic approach of rehabilitation, cancer rehabilitation in fact should comprise multidisciplinary efforts including, among others, medical, psychological and physiotherapeutic treatment as well as occupational therapy and functional therapy, depending on the patient’ s functional status [55,56]. Maintaining continuity, through coordination, represents one aspect of rehabilitation in which nursing has a key role that has been widely addressed in oncology nursing literature.

We believe that our findings have the potential to make a contribution to the development of rehabilitation nursing and that this training course, the first of this kind in Italy, could be incorporated into undergraduate nursing education programmes and also be inserted into continuing education programmes for graduate nurses. However further research is needed to refine the contents of the teaching units and to evaluate its feasibility and costs. The content of the entire curriculum is in fact open to modification on the basis of evaluations and feedback after the first implementation.

Professional rehabilitation nurses must, in fact, combine their practice with continuing education in order to acquire specific knowledge and skills that will contribute to more efficient rehabilitation processes and services.

By teaching registered nurses the principles of rehabilitation nursing, and creating, for them, the specific qualification of neurorehabilitation nurse, the quality of overall care for neurological patients could be improved, through fewer complications, shorter hospital stays, better and outcomes and better support for families.

Recent studies reported that the presence of nurses with higher educational level improves patients’ outcomes. In fact, although it has not been conclusively demonstrated the link between the level of training and quality of care, associations between a series of patients’ outcomes, including mortality, and the training of nurses are well documented [57,58].

Developing expertise in neuro-rehabilitation for nurses, will be critical to improve overall care according to the “simultaneous care” model [59] particularly for patients affected by BT, for which the integration of different professionals expertise can provide solutions to the complex needs of the patient and caregivers [60,61].

In this view, nurses can contribute to the quality and satisfaction of patients’ lives by developing a philosophy that incorporates rehabilitation principles as integral part of their practice.

Nursing profession has already made a significant contribution to the body of knowledge in the field of rehabilitation of the cancer patients and his/her family; new generations of allied health professionals need a solid grounding in clinical skills, but as already suggested by previous authors, they also need a strong educational background and attitudes that will enable them to build their profession as well as their own professional practice [62,63]. These attitudes and skills have been suggested to include a desire to engage in lifelong learning and professional growth and an ability to identify and critically evaluate their own practice and the underlying theories and perceptions that inform the practice of nursing [64].

In our view, the crucial next step will be to start discussing, at the level of scientific societies linked to the field of neurorehabilitation and oncology, the development of a specialisation course in neurorehabilitation nursing.

## Competing interests

The authors declare that they have no competing interests.

## Authors’ contributions

MB conceived the paper, interpreted data and wrote the final manuscript; CZ conceived the paper, interpreted data and wrote the final manuscript; AP reviewed and commented the last version of the manuscript; AMDN helped to revise the first draft of the manuscript; MS and GS reviewed and commented the last version of the manuscript; FP interpreted data, reviewed and commented the last version of the manuscript. All authors read and approved the final manuscript.
